# Silver Nanowires in Stretchable Resistive Strain Sensors

**DOI:** 10.3390/nano12111932

**Published:** 2022-06-06

**Authors:** Srinivasan Raman, Ravi Sankar Arunagirinathan

**Affiliations:** 1 School of Electronics Engineering (SENSE), Vellore Institute of Technology (VIT), Chennai, Tamil Nadu 600127, India; srinivasan.r2019@vitstudent.ac.in; 2 Centre for Innovation and Product Development (CIPD), Chennai Campus, Vellore Institute of Technology (VIT), Chennai, Tamil Nadu 600127, India

**Keywords:** AgNW, silver nanowires, stretchable strain sensors, wearable sensors, metal nanowires, human motion monitoring

## Abstract

Silver nanowires (AgNWs), having excellent electrical conductivity, transparency, and flexibility in polymer composites, are reliable options for developing various sensors. As transparent conductive electrodes (TCEs), AgNWs are applied in optoelectronics, organic electronics, energy devices, and flexible electronics. In recent times, research groups across the globe have been concentrating on developing flexible and stretchable strain sensors with a specific focus on material combinations, fabrication methods, and performance characteristics. Such sensors are gaining attention in human motion monitoring, wearable electronics, advanced healthcare, human-machine interfaces, soft robotics, etc. AgNWs, as a conducting network, enhance the sensing characteristics of stretchable strain-sensing polymer composites. This review article presents the recent developments in resistive stretchable strain sensors with AgNWs as a single or additional filler material in substrates such as polydimethylsiloxane (PDMS), thermoplastic polyurethane (TPU), polyurethane (PU), and other substrates. The focus is on the material combinations, fabrication methods, working principles, specific applications, and performance metrics such as sensitivity, stretchability, durability, transparency, hysteresis, linearity, and additional features, including self-healing multifunctional capabilities.

## 1. Introduction

Flexible and stretchable strain-sensing composites are gaining popularity in human motion monitoring, healthcare, intelligent textiles, robotics, and structural health monitoring [1]. Since conventional strain gauges made of metal foils and semiconductor materials have limitations in terms of sensitivity and stretchability, alternative flexible and stretchable materials are continuously explored. To this end, various polymer composites have been developed to make flexible and stretchable strain sensors [2], which are classified into piezoresistive, capacitive [3], piezoelectric [4], triboelectric [5], optical [6], and fiber Bragg grating [7] strain sensors based on the principle of operation. Piezoresistive sensing has the advantages of simple fabrication methods, low power consumption, and a wide sensing range. Stretchable piezoresistive (or resistive) strain sensors are often realized using electrically conductive polymer composites with sensing elements deposited on flexible and stretchable support materials [8]. Sensing elements or functional materials, or fillers are electrically conductive. They are classified into carbonaceous materials (e.g., graphene variants and carbon nanotube (CNT) variants (single-walled and multi-walled), carbon nanofibers (CNFs), carbon black (CB, etc.), metal nanostructures (e.g., silver nanowires (AgNWs), silver nanoparticles (AgNPs), gold nanowires (AuNWs), copper nanowires (CuNWs, etc.), intrinsically conducting polymers (e.g., polyaniline, polypyrrole, poly(3,4-ethylenedioxythiophene) polystyrene sulfonate (PEDOT:PSS), etc.), ionic liquids, liquid metals, and MXenes (e.g., Ti_3_C_2_T_x_) [9]. Metal nanowires such as AgNWs have high electrical conductivity, flexibility, solution processability, and transparency, facilitating the superior sensing performance of sensors [10].

As for flexible and stretchable strain sensors, many review articles can be found in the literature. For example, Amir Servati et al. presented a review on novel flexible and wearable electronic materials and sensors suitable for monitoring vital human signs [11]. Yan Liu et al. [12] and Fei Han et al. [13] reviewed the advances in flexible strain sensors, focusing on materials, mechanisms, applications, and manufacturing strategies. Few other reviews elucidate the progress in this type of sensor from the angle of filling elements, i.e., carbon-based nanomaterials [14], metal nanowires [15], and conducting polymers [16]. Further, there are review articles giving insights into AgNW-based composites, with a focus on fabrication methods and typical applications [17,18], properties [19], and specific applications, such as optoelectronics [20], energy devices [21], organic electronics [22], and flexible electronics [23]. Recently, Amit Kumar et al. [24] reported a detailed review of synthesis methods and strategies for AgNW-based transparent conductive electrodes and various treatment methods to improve their optoelectronic properties. A review by Neha Sharma et al. [25] focused on recent developments in AgNW-based composites used in various sensors, displays, and energy devices. However, to the best of our knowledge, no review article reported to date exclusively focuses on stretchable strain sensors based on AgNW fillers.

This review article discusses the recent developments in AgNW-based stretchable strain sensors grouped according to their substrates. A brief introduction and applications of resistive stretchable strain sensors and AgNW–polymer composites are given in Section 2 and Section 3, respectively. Then, a substrate-wise analysis is presented in the following sections. Polydimethylsiloxane (PDMS), thermoplastic polyurethane (TPU), and polyurethane (PU) are widely used substrates to form polymer composites with AgNWs. In Section 4, an update on AgNWs as a single filler (Section 4.1) or additional filler (Section 4.2) in PDMS-based stretchable strain sensors is given. Section 5 and Section 6 cover the developments in AgNW/TPU- and AgNW/PU-based stretchable strain sensors, respectively. Section 7 details stretchable strain sensors made of AgNWs and other substrates, such as cellulose film, Ecoflex, Dragon Skin (DS), and natural rubber. In each section, the conducting element/polymer combination, fabrication methods, sensitivity, working strain range (stretchability), durability, applications, and additional features are tabulated.

## 2. Stretchable Resistive Strain Sensors

Stretchable resistance-based strain sensors work on the principle of change in resistance to physical deformation. Resistance changes are based on disconnections, crack propagations, and tunneling effects [26]. They, in general, consist of one or more layers of stretchable polymer matrix or a fibrous material consisting of one or more electrically conducting nanomaterials. These types of sensors display superior performance compared to conventional strain gauges. The list of substrates includes PDMS [27], PU [28], TPU [29], Ecoflex [30], poly(styrene-butadiene-styrene) (PSBS) [31], Dragon Skin [32], cotton/spandex [33], etc. The conducting elements can be carbon-based nanomaterials, metal nanoparticles, metal nanowires, conducting polymers, MXenes, liquid metals, ionic liquids, etc. Fabrication methods such as drop casting, spin coating, dip coating, vacuum filtration, inkjet printing, and spray coating are widely used to prepare stretchable resistive conducting composites [34].

### 2.1. Operation Mechanisms

**Figure 1:**
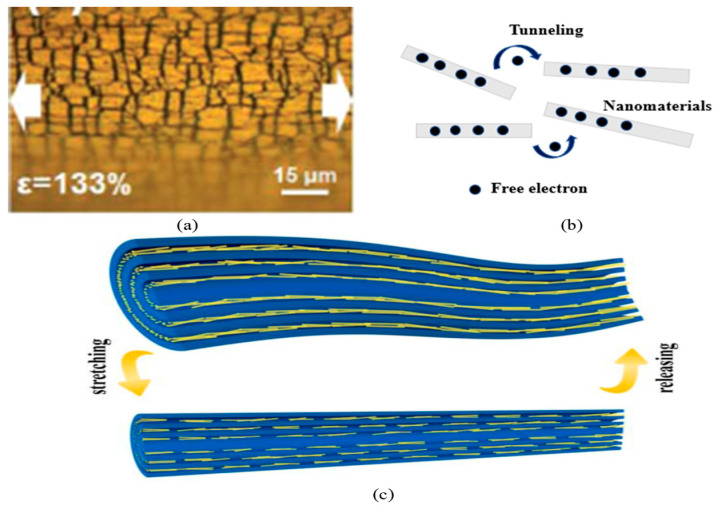
(**a**) Atomic force microscope image of crack formation on Au thin-film-coated PU cord. Reprinted with permission from Ref. [40]. Copyright 2017, Royal Society of Chemistry. (**b**) The transition of free carriers among conducting materials through the non-conducting barrier. (**c**) A schematic to illustrate disconnection and reconnection of nanowires in conductive fibers on stretch and release, respectively. Reprinted with permission from Ref. [27]. Copyright 2021, Elsevier.

The three primary mechanisms by which the increase in resistance upon the application of strain is achieved are crack propagation, disconnection/reconnection, and tunneling effects [13]. In the case of strain sensors with a nanomaterial thin film coated on a flexible substrate, the principal mechanism of the increase in resistance is based on the propagation of cracks [35]. When the conducting materials form a thin layer on a flexible substrate, cracks are generated in stress-concentrated areas. As shown in Figure 1a, the opening and enlargement of the cracks limit the electrical conduction paths in the film and increase the resistance of the strain sensor under strain. Cracks gradually propagate perpendicular to the stretching direction, breaking the nanowire–nanowire junction in the composite. Most of the generated cracks close when the applied strain is removed, and the initial morphology is almost recovered [36]. In a few sensors, controlled crack propagation has been used to realize the enhanced sensitivity of stretchable strain sensors [37,38]. The microcrack-based sensors’ sensitivity is generally high, as the resistance variation is high during stretching compared to other sensors without microcracks [39]. Crack-based sensors with high sensitivity can be applied to feeble strains, such as muscle motions and vital activity [12]. Pre-straining is a method for installing cracks [40]. The crack density decreases with increasing pre-strain. A sensor with more cracks generates a larger resistance variation [41]. The dimensions, depth, and position of cracks influence the sensing performance and the working range of crack-based sensors. In most cases, the crack propagation mechanism is accompanied by the tunneling mechanism because few carriers can still hop through adjacent points [35].

Another mechanism involved in the resistance variation is the disconnection, as shown in Figure 1c, among nanomaterials forming conducting paths. Electrons pass through conduction paths formed by the overlapping of nanomaterials within the percolation network. With large strains, overlapping nanomaterials tend to disconnect, as they have a lower elongation at break and a higher Young’s modulus than the elastomer [26]. The slippage phenomenon is prevalent in nanowires and flake-based conducting networks. In the case of AgNW networks, adjacent nanowires also slip and separate from each other, resulting in partial disconnection, leading to increased resistance [27]. Tunneling is the crossing of electrons through non-conductive barriers, as shown in Figure 1b. Within a certain cut-off distance between nanomaterials, electrons can hop between them through non-conductive thin layers forming quantum tunneling junctions and direct electrical conduction paths [1]. According to Simmon’s theory, the tunneling resistance between nanomaterials depends on the distance between nanomaterials, the cross-sectional area of the tunnel, and the height of the energy barrier. When strain is applied, the cut-off distance and hence the tunneling resistance change [35]. Repeated static and cyclic loadings or overstretching can cause permanent disconnection and the irreversible loss of conduction paths, which inhibit the reliability and accuracy of the strain sensor [26].

### 2.2. Characteristics and Applications

The performance of a resistive-type strain sensor is evaluated by the sensitivity (gauge factor (GF)), strain range (stretchability), durability, low detection limit (resolution), response time, linearity, and hysteresis [42]. Sensitivity or GF is the ratio of the change in resistance to the change in the length of the sensing element. The low detection limit is the minimum strain that the sensor can detect, and stretchability is the highest strain that it can measure. It is often challenging to obtain high sensitivity and a wide strain range in the same sensing element. Durability is the number of stretch/release cycles in which the sensor’s electromechanical response is the same as the initial response. High sensitivity, high stretchability, low resolution, fast response time, high linearity, and low hysteresis are the desired features of any stretchable strain sensors. In recent times, self-healing ability [43], biocompatibility [44], and multifunctional sensing [45] have become a few additional features realized with these sensors.

Due to their flexibility and stretchability, resistive-type electrically conductive polymer composites show great potential in motion monitoring, artificial muscles, human–machine interfaces (HMIs), soft robotics, etc. [46]. These resistive-type sensors can sense vital signals, such as pulse and respiration rate [47]. Minor strains detected by these sensors include facial expressions, coughing information, saliva swallowing motion, and vocal cord vibrations [48]. Body part movements such as finger bending are suitable in sign language recognition [49], and joint movements such as the knee, ankle, elbow, wrist, etc., are used in elderly care, gait analysis, rehabilitation processes, and sports performance analysis [50]. Besides biomedical applications, stretchable strain sensors are widely used in stress monitoring and crack detection in structures like buildings, bridges, airplanes, parachute canopies, windmill blades, engines, etc.

## 3. AgNWs in Polymer Composites

**Figure 2:**
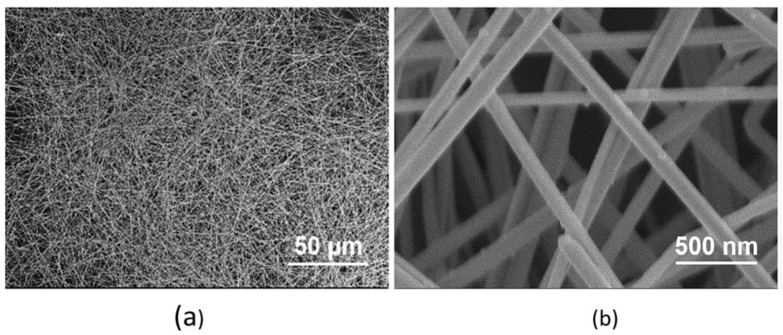
Microlevel images of long AgNWs prepared by the one-step polyol method (**a**) at 50 µm scale and (**b**) at 500 nm scale. Reprinted with permission from Ref. [59]. Copyright 2019, Royal Society of Chemistry.

Metal nanowire networks can be prepared using fast, facile, and solution-processed approaches. They possess high intrinsic electrical and thermal conductivity and are flexible, mechanically robust, and low-cost. Ag is one of the most electrically conductive bulk materials at room temperature (15.87 × 10^−9^ Ω·m at 20 °C) [51]. Silver has many nanomaterial forms, which include Ag powder [52], Ag nanoparticles [53], Ag ink [54], Ag nanosheets [55], and Ag nanowires [56]. Silver nanowires are prepared using UV irradiation, hydrothermal, photoreduction, template-based, wet chemical, and solution-based synthesis methods [57]. Compared to other categories of fillers, AgNWs are more flexible, transparent, electrically conducting, solution-processable, and compatible with a variety of substrates. Silver nanoparticles and silver nanowires are other potential elements for enhancing the sensitivity of strain sensors [58]. The length and diameter of the AgNW decide the aspect ratio, which impacts the stability of the strain sensor. It is desirable that the aspect ratio of the AgNW is higher, and it can vary from a few hundred to a few thousand. A typical scanning electron microscopic (SEM) view of AgNWs is shown in Figure 2.

**Figure 3:**
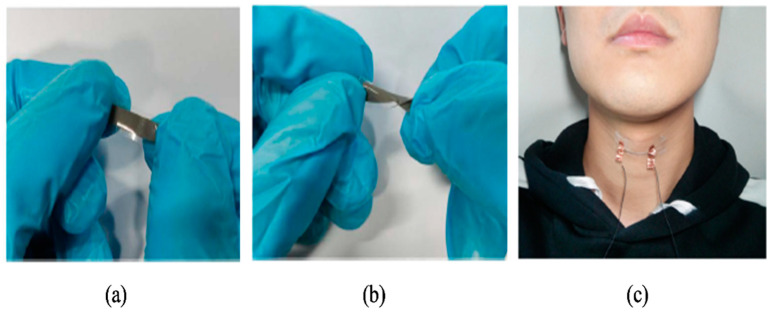
Images of AgNW composites to show (**a**) stretchability and (**b**) bendability. Reprinted with permission from Ref. [56]. Copyright 2020, Wiley Online Library. (**c**) Attachment of AgNW/PU strain sensor to the throat to sense strain. Reprinted with permission from Ref. [28]. Copyright 2019, American Chemical Society.

Due to their transparency, low sheet resistance, low cost, solution processability, and compatibility with different substrates, AgNWs are emerging as an alternative to indium-doped tin oxide (ITO) in new-generation photovoltaics [51]. Silver nanowire networks show enhanced performance in organic electronics that cover light-emitting diodes, photovoltaic cells, transistors, and memory devices [22]. As transparent conducting electrodes (TCEs), they are part of several applications, such as optoelectronic devices [20], electrochemical devices [60], and energy devices [21]. They are applied in surface-enhanced Raman scattering (SERS) [61] and EMI shielding [62] as well. As conducting elements in stretchable strain sensors, they are used only as conducting elements or as additional filler in the matrix to enhance the sensitivity and the sensing range. The AgNW networks of the composite form electrically conducting paths in the relaxed state. Upon stretching, the gap between the nanowires gradually increases, reducing the connections and increasing the resistance in the path. The bendability and stretchability of AgNW composites (Figure 3a,b) enable applications such as human motion monitoring (Figure 3c).

## 4. AgNW/PDMS-Based Stretchable Strain Sensors

PDMS is the mainstream substrate often used to prepare stretchable strain sensors. In preparing the PDMS elastomer, the base monomer is added with the curing agent in the 10:1 ratio. Silver nanowires have been paired with PDMS to implement stretchable strain sensors with varied strain ranges and sensitivity. This section describes the recent developments in preparing stretchable strain sensors using AgNWs as a single filler (Section 4.1) and additional filler (Section 4.2) in the PDMS composite.

### 4.1. AgNWs as a Single Filler in Strain-Sensing PDMS Composites

In a few research works, AgNWs were used as the only filler in the PDMS substrate, but the novelty was introduced via fabrication steps. In the early stages, Morteza Amjadi et al. reported a sandwich model nanocomposite with AgNW thin film in between two layers of PDMS using the drop-casting method [63]. The composite displayed a stable response, good linearity, low hysteresis, and response to bending. The resistance change is based on the disconnection mechanism between AgNWs and topological changes in the network. In another instance, micropatterned electrodes were fabricated by Hyungdong Lee et al. using dispensing nozzle printing of an AgNW/PDMS composite. The number of fillers was related to the liquid ejection time, and the electrical resistance varied with printing speed [64]. With stretchability of up to 60%, the electrodes obtained by this printing method were suitable for electronic skin. In another work, tunable strain sensors based on 2D AgNW networks were implemented by Xinning Ho et al. The sensitivity depends on the surface coverage, which is determined by the volume of the AgNW solution and the waviness of the AgNWs [65]. Waviness is established in the nanowire network when the PDMS substrate is transfer-printed with vacuum-filtered AgNWs.

**Figure 4:**
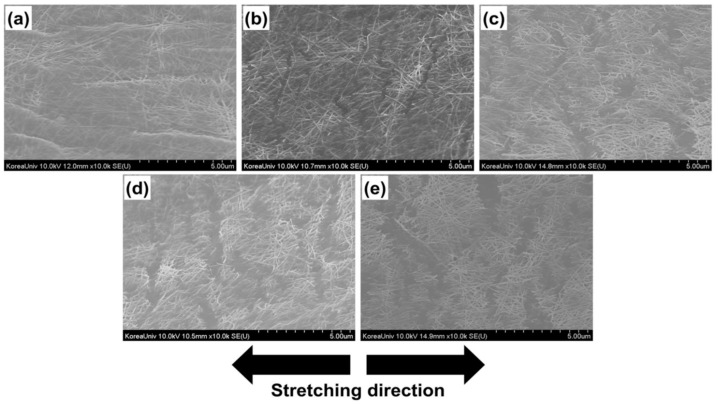
SEM images of AgNW/PDMS (**a**) in the relaxed state and at a strain of (**b**) 25%, (**c**) 50%, (**d**) 75%, and (**e**) 100%. Adapted under the terms of the CC-BY 4.0 license from Ref. [67]. Copyright 2017, The Authors, published by Nature Portfolio.

AgNW patterns can be directly formed on various substrates on a wafer-scale using a parylene stencil process. In the work of Namsun Chou et al., parylene was coated on surface-treated PDMS first and patterned using lithography and reactive ion etching. Then, AgNWs were spray-coated, and the parylene was peeled off. Using the as-prepared AgNW electrodes, resistive strain sensors to measure deformation and capacitive tactile sensors to gauge pressure can be realized. The fabricated sensors can sense various minute and large strain signals [66]. Crack-based strain sensors are highly sensitive but, at the same time, limited by stretchability. In Chan-Jae Lee et al., AgNWs dispersed in isopropanol were spin-coated on a PDMS film, and then the sensor was stretched and released at a particular strain to form cracks in the structure, as shown in Figure 4 [67]. Polyurethane urea (PUU) encapsulates the AgNW/PDMS composite for mechanical stability. PUU enhances AgNW and PDMS adhesion to attain high sensitivity and stretchability. The PUU layer is transparent and stretchable, and it helps the percolated Ag network with ample conducting paths in response to stretching.

**Figure 5:**
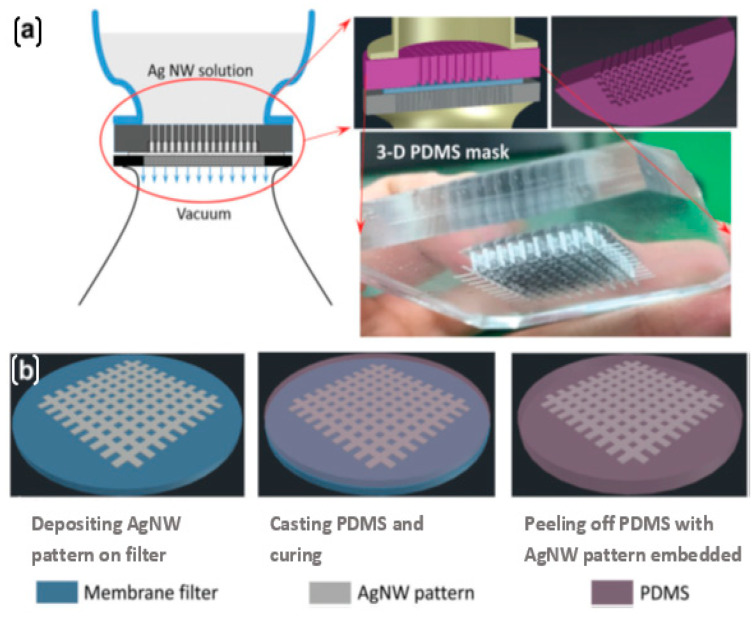
Steps in fabricating AgNW patterns on a membrane filter using 3D mask (**a**) Vacuum filtration the process (**b**) Depositing AgNW pattern, transferring to target PDMS substrate, and peeling off PDMS. Reprinted with permission from Ref. [68]. Copyright 2018, Royal Society of Chemistry.

The conventional 2D mask method for making AgNW patterns has limitations in preparing complex patterns. A 3D mask, in combination with a filtration system, as shown in Figure 5, allows efficient manufacturing of complex AgNW patterns with precise edges [68]. The applied vacuum aids in the adherence of the 3D mask’s bottom surface to the membrane layer. The AgNW solution is deposited solely on the desired area of the membrane filter after flowing through channels inside the 3D mask. AgNW patterns of various grid shapes with defined borders were manufactured with high efficiency. A strain range of over 80% and tunable gauge factors ranging from 0.07 to 520 were achieved by adjusting the AgNW deposition density and the PDMS peel-off direction. The electrical resistance decreased as the AgNW deposition density increased.

**Figure 6:**
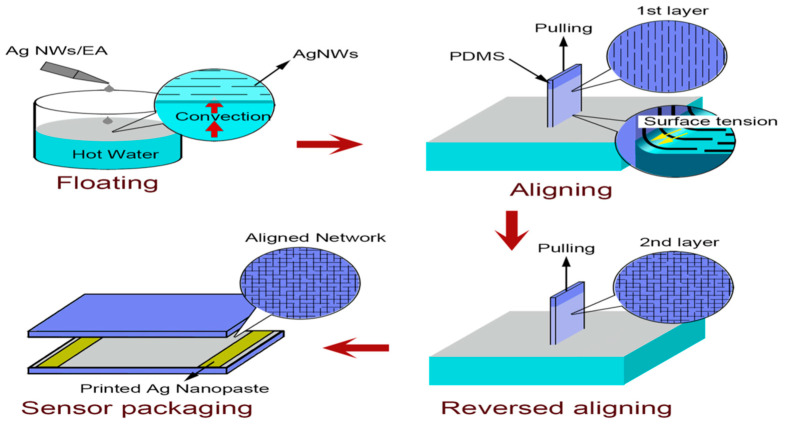
Fabrication steps in the water-bath pulling method. Adapted under the terms of the CC-BY 4.0 license from Ref. [69] Copyright 2019, The Authors, published by Nature Portfolio.

An ordered AgNW array on PDMS substrate achieves higher sensitivity and transparency. Strain sensors fabricated using an ordered AgNW array/PDMS composite and a simple water-bath pulling method displayed a GF of 84.6 and a transparency of 86.3% [69]. AgNW ohmic contacts are formed when the PDMS surface is pulled out of the solution in two orthogonal directions, as shown in Figure 6.

The alignment of AgNWs also impacts the strain sensor performance, and it can be controlled. As reported in the works of Jae Hyuk Choi et al., longitudinally aligned strain sensors demonstrate a narrow strain range (ε < 25%) and high GF (89.99). In comparison, laterally aligned strain sensors exhibit relatively low sensitivity (GF < 22.10) and a high strain range (ε < 60%) [70].

AgNWs were prepared using the modified polyol method in most of the research works reported. An improved polyol method to prepare AgNWs in 30 min is described in the work of Wei Li et al. [58]. The reaction temperature, the molecular weight of polyvinyl pyrrolidone (PVP), the ratio of silver nitrate (AgNO_3_), and PVP affect the sensing characteristics. The flexible, stretchable AgNW@PDMS sensor prepared by this semi-dry method had good stability and sensitivity and low hysteresis and was tested for joint movements. Wei-Wei Kong et al. reported a fibrous strain sensor made of a rolled-up PDMS sheet spray-coated with AgNWs [27]. The fiber’s cross-section contained spirals (or rings) similar to the growth rings of a tree. The AgNW/IPA dispersion is spray-coated onto an O_2_ plasma-treated PDMS film. Copper foils are connected as electrodes to the film by silver coating paint. Finally, the AgNW/PDMS composite film is rolled up manually, and a liquid PDMS mixture seals the edge. Increasing the spray volume reduces the electrical resistance as the overlapping AgNWs increase. Upon stretching, the adjacent AgNWs slip and separate from each other, resulting in partial disconnection of the conducting network and increased resistance. Table 1 shows the overview of strain sensors made using AgNWs as the only filler in the PDMS composite.

### 4.2. AgNWs as Additional Fillers in PDMS Composites

To obtain superior sensing characteristics, more than one filler is added to the substrates. Hybrid fillers contribute to the performance through the synergy between them. For instance, strain sensors using Ag nanomaterial samples containing AgNWs and AgNPs synthesized using FeCl_3_ solution as the growth control agent via the heat polyols thermal method exhibit a high sensitivity of 547.8 [74]. Percolating networks of thin gold nanowires (AuNWs) and rigid silver nanowires were employed to fabricate transparent wearable sensors [75]. The combination of soft AuNWs and more rigid AgNWs enables the production of strain sensors suitable for biometric information collection, facial expression detection, and respiration and apexcardiogram monitoring.

**Figure 7:**
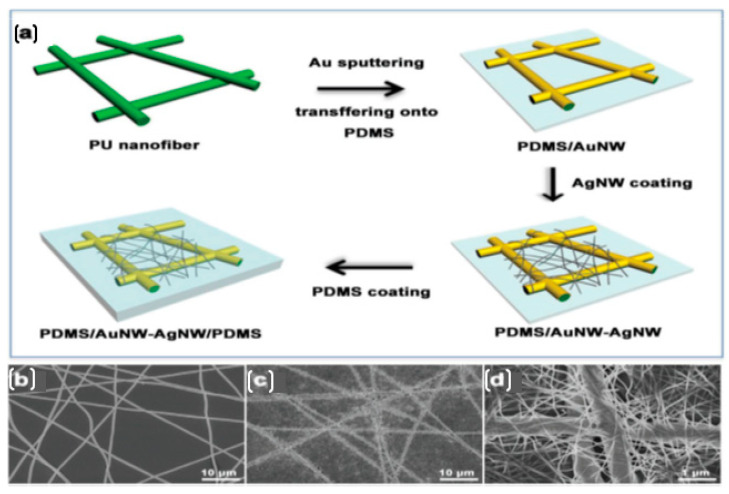
(**a**) Schematic illustrations of stretchable AgNW-AuNW/PDMS composite fabrication steps. Top-view EM images of (**b**) AuNWs and (**c**,**d**) AuNW–AgNW hybrid networks on PDMS substrate. Reprinted with permission from Ref. [76]. Copyright 2018, Wiley Online Library.

Similarly, Shasha Duan et al. [76] reported a binary hybrid network of small-sized AgNWs and a continuous AuNW backbone. The schematic illustration of fabrication steps is given in Figure 7. In the low strain range, the AgNW percolation network provides considerable sensitivity via the disconnection mechanism, whereas the AuNWs serve as connectors between isolated AgNW regions in the increased strain range. These invisible, wearable, and stretchable electrodes successfully recorded activities such as smiling, finger bending, and knee bending.

**Figure 8:**
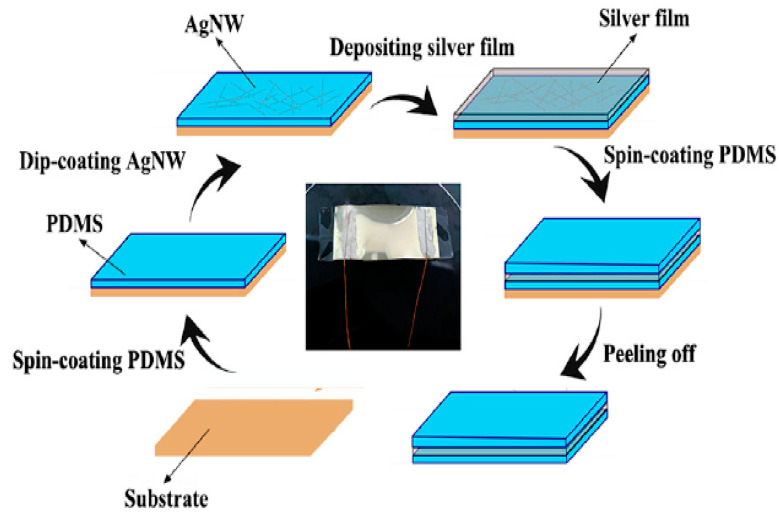
Schematic illustration of steps involved in fabricating strain sensors by deposition of the silver film. Adapted under the terms of the CC-BY 4.0 license from Ref. [77] Copyright 2019, The Authors, published by MDPI.

A combination of Ag film and AgNWs as fillers in PDMS film was experimented with by Jinjin Luan et al. [77]. In the fabrication process, as shown in Figure 8, PDMS film is dip-coated in a dispersion of AgNWs, followed by a 100 nm thick Ag film deposition by vacuum thermal evaporation. While most AgNWs are well inside the Ag film, the junctions of intersecting nanowires pop out of the Ag film. The film’s Ag particles around the exposed AgNWs improve the conductivity by increasing the contact surface area. A spin-coated PDMS film on top of the Ag film prevents the oxidation of the same and improves the lifetime of the sensor.

In a different attempt, Shahid Aziz et al. [78] reported a stretchable strain sensor using 3D zinc stannate (ZnSnO_3_) nanocubes and 1D AgNWs in a PDMS elastomer. The performance characteristics were determined by mixing ratios of the nanocubes and nanowires. The high-aspect-ratio Ag-NWs increased the distribution of ZnSnO_3_ nanocubes in the PDMS matrix and reduced the total internal resistance of the ZnSnO_3_/PDMS composite. The sensor demonstrated a sensitivity of 26.7 kΩ/ε and stretchability of 100% with durability of more than 10,000 cycles. Multifunctional wearable devices containing strain-sensing elements have attracted the attention of researchers. Ge Shi et al. developed a strain-sensing and drug-delivering system on an elastic dry-adhesive substrate [45]. The strain sensor had graphene nanoplatelets (GnPs) and an AgNW composite as the sensing materials, which were deposited layer by layer through vacuum filtration. An AgNW composite-based strain sensor developed via different ultrasonication-based patterning showed high transparency and high sensitivity with a broad strain range [79]. The AgNW acrylate composite is UV cross-linked to produce a brittle layer for crack development at tiny strains, and the AgNWs form hydrogen bonds with the substrate for improved stability.

Carbon fillers have also been additionally paired with AgNWs. Combining the superior conductivity of AgNWs and brittle layers provided by graphene for sensing, a stretchable sensing film embedded in two PDMS layers was fabricated. AgNWs, graphene, and AgNWs were vacuum-filtered in sequence, followed by the injection of liquid metal as electrodes [80]. The inner graphene slips under tiny strains, and the outer AgNWs disconnect under larger strains, enabling a sensing range of 0–35% and a GF of 111.5 at 1% strain. The volume and timing of each filtration can tune the attributes of the sandwich-based strain sensor. Incorporating the Ag nanowire/graphene (AgNW/G) composite into the PDMS polymer allows strain sensor flexibility even at low temperatures and low hysteresis [81]. The sensor showed anti-interference ability against temperature in the 0−24% strain range. In the fabrication process, graphene nanosheets are dispersed in an AgNW ethanol solution to obtain the suspension of AgNW/G. Then, the AgNW/G suspension is drop-coated three times onto the surface of semi-solidified PDMS to obtain a uniform distribution of the AgNW/G composite on the PDMS surface. An overview of recent research works where AgNWs are the additional filler in the strain-sensing composite is shown in Table 2.

## 5. AgNW/TPU-Based Stretchable Strain Sensors

**Figure 9:**
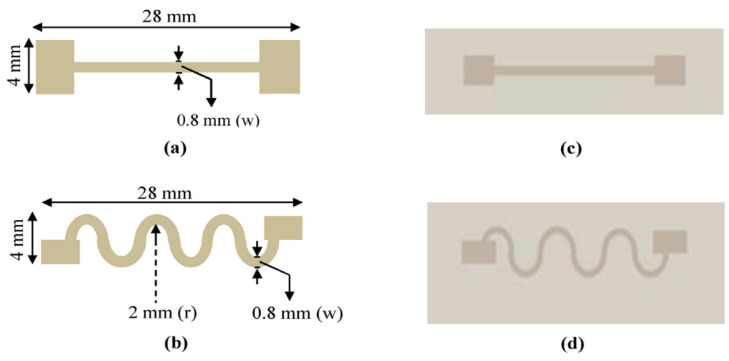
Schematic of (**a**) straight-line and (**b**) wavy-line configurations of the strain sensor. Images of screen-printed (**c**) straight- and (**d**) wavy-line configuration-based strain sensor TPU substrate. Reprinted with permission from Ref. [87]. Copyright 2018, Elsevier.

Thermoplastic polyurethane is dissolved in solvents such as dimethylformamide (DMF) and anhydrous tetrahydrofuran (THF) before forming an elastomeric base by casting or a fibrous substrate by electrospinning. Research works relating to AgNWs as fillers in a TPU substrate are summarized in this section. For detecting microstrains, such as pulse beat detection, and sounds, a cracking-assisted AgNW/graphene hybrid/TPU sensor was fabricated by Song Chen et al. [86] using simple co-precipitation, reduction, vacuum filtration, and casting. The crack and overlap structure are formed by pre-stretching, and the sensor exhibits GFs as high as 20 (for strain ε < 0.3%), 1000 (0.3% < ε < 0.5%), and 4000 (0.8% < ε < 1%). M M Ali et al. implemented strain sensors by screen printing AgNW/Ag flakes onto the TPU substrate in two configurations (straight line and wavy), as shown in Figure 9. The strain range was only 0 to 10%. Still, they achieved gauge factors of 22 and 33 for straight-line and wavy configurations, respectively [87].

Highly stretchable, electrically conductive, and transparent films suited for wearable electronics and health monitoring were developed by Runfei Wang et al. by using AgNW/TPU [88]. The AgNW solution was rod-coated onto a glass slide, and TPU solution was poured onto the AgNW film. Then, the AgNW/TPU layer was detached from the glass substrate. As the thickness of the film increased, transparency decreased.

A thermoplastic polyurethane electrospun membrane (TPUEM) vacuum filtered with an AgNW conductive network, followed by spin-coating PDMS, could function as a flexible and stretchable strain sensor [89]. In another instance, an electrospun porous TPU membrane was immersed in the AgNW solution to develop an AgNW/TPU-based stretchable strain sensor [29]. By varying dip-coating cycles, the content of AgNWs and the conductivity of the nanomembrane are adjusted. A spring-like configuration with neat loops obtained using a rotating device enhances the stretchability to 900%. By layer-by-layer spray coating of the AgNW solution and GO solution onto flexible electrospun TPU fibrous mats, a flexible and stretchable strain sensor was developed by Yan Li et al. [90]. With the lowest value of 450, the GF varied depending on the strain range of strains sensed. Due to the synergistic effect of AgNWs and rGO, high stretchability and sensitivity were achieved. The sensitivity and sensing strain range can be varied by regulating the volume ratio of AgNWs and rGO.

In an attempt to achieve low-resistance, mechanically stable, and breathable composites, a network of AgNWs was sandwiched between two highly porous electrospun TPU membranes [91]. The membranes were robust to both bending and stretching, and they had an elongation at break of 700%. Dispersing short fibers of polycaprolactone (PCL) in the AgNW network improved the interface stability. The membranes were breathable, allowing the exchange of gases for human comfort. Stretchability as high as 565% and a GF as good as 6886 were made possible by the aid of materials such as tannic acid (TA) and hydrolyzable 3-aminopropyltriethoxysilane (APTES) hybrid coating in depositing AgNPs onto the TPU substrate [92]. Table 3 summarizes recent works relating to stretchable strain sensors made of AgNW/TPU composite.

## 6. AgNW/PU-Based Stretchable Strain Sensors

Composite films can be prepared with PU as the stretchable substrate and AgNWs as the conducting element [28,59]. PU fibers are also part of the latest electronic sensors and intelligent fabrics, as they are lightweight, flexible, and knittable. The fibers’ electrical conductivity increases with the number of coating cycles [28]. At the same time, the percolation threshold of the composite can be reduced by improving the dispersion of fillers. In a study by Yong Wei et al. [49], a paper-based bending sensor with AgNWs and 2D Co−Al layered double hydroxide (LDH) nanosheets in waterborne polyurethane was reported. The 2D LDH nanosheets were embedded into the AgNW network to assist the dispersion of AgNWs. The conductive composites had a low percolation threshold and can be manufactured via various printing methods. The bending sensor from this composite showed durability of more than 3000 cycles, a sensitivity of 0.16 rad^−1^, a response time of 120 ms, and a relaxation time of 105 ms.

Pre-straining is a method to extend the stretchability of the AgNW conductive-networked PU cord [40] without adding other structural materials such as cotton yarns. Optimized pre-strain conditions and nanowire density yield the cord with the best stretching performance. AgNWs can be embedded in PU fibers by the capillary tube method to form completely conductive networks [28]. A capillary glass tube (CGT) is first immersed in an AgNW suspension in the fabrication process. AgNW networks are formed in the CGT as the suspension moves inside to the other end. After drying, polyurethane is drawn into the CGT by the negative pressure. The glass is removed by etching using HF, and the PU/AgNW fiber is obtained. AgNW-treated PU nanofibers can also be coated with a PDMS layer to enhance durability to function as sensors for joint flexion monitoring [93].

As an additional filler in PU-based stretchable strain-sensing composites, AgNWs enhance the performance metrics obtained with other fillers. In Jun-Hong Pu et al., AgNW/WPU and MXene layers were alternatively and firmly coated onto a hydrophilic polyurethane-based commercial fiber (HPUF) utilizing a water solution-based layer-by-layer dip-coating process to create homogeneous and stable sensing layers. AgNW/WPU layers in the structure preserve the sensing layer’s integrity at high strain, whereas MXene layers efficiently encourage fracture development across the whole operating range [94]. A dual-parameter sensor that can transduce both temperature and strain into electrically isolated signals was developed by Fengchao Li et al. using printable titanium carbide (MXene)-silver nanowire (AgNW)-PEDOT:PSS-tellurium nanowire (TeNW) nanocomposite in a multi-level hierarchical architecture. The sensing devices were fabricated by depositing nanocomposite gel onto an O_3_ plasma-treated polyurethane substrate [95]. The synergistic effects between all nanomaterials enhance the stretchability and sensitivity. The crack propagation effect of the conductive MXene-AgNW network and thermoelectric effect of the TeNW-PEDOT:PSS network can sense the strain and thermal stimulus, respectively. With the inclusion of PEDOT:PSS, the strain range is expanded to over 60% from 40% at the expense of sensitivity. However, the amalgamation of MXene, AgNWs, PEDOT:PSS, and TeNW improves both the sensitivity and stretchability. An overview of a few research works relating to AgNW/PU strain-sensing composites is given in Table 4.

## 7. AgNW/Other Substrate-Based Stretchable Strain Sensors

This section discusses the methods of AgNW incorporation into substrates other than PDMS and TPU and the resulting composites’ performance characteristics. First, composites with AgNWs as the only filler are discussed, and then composites with AgNWs as the additional filler are discussed.

### 7.1. AgNWs as the Only Filler in Other Substrate-Based Strain Sensors

**Figure 10:**
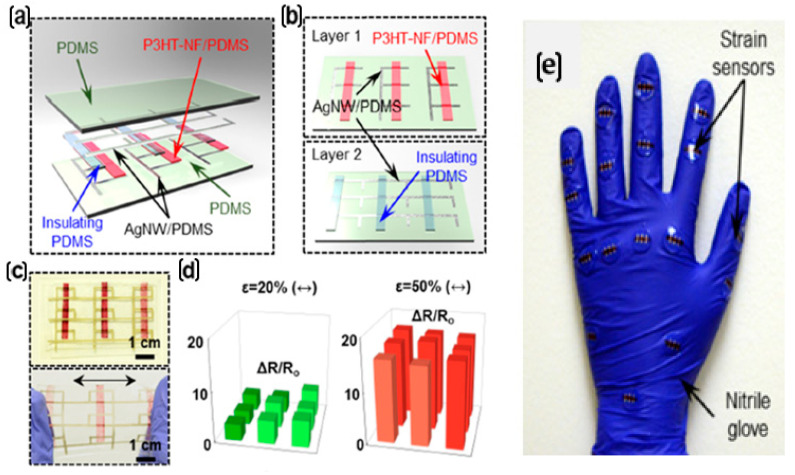
(**a**) A 3 × 3 strain sensor array. (**b**) Schematic of layers 1 and 2. (**c**) The array in the normal state (upper) and stretched state (lower). (**d**) The ΔR/R_o_ of the array upon 20% and 50% mechanical strain. (**e**) Strain sensors placed on a hand glove for gesture recognition. Reprinted with permission from Ref. [99]. Copyright 2018, American Chemical Society.

Recent developments in stretchable strain sensors using various other substrates are described in this section. A microprism-structure-based strain sensor using AgNW/Dragon Skin composite was reported by K H Kim et al. [32]. A silicon master micropatterned by soft lithography is coated with AgNWs using the drop-casting process. Then, a liquid pre-polymer of Dragon Skin (DS) mixed with a curing agent is poured onto the AgNW-coated silicon master. While the metal nanowire percolation network forms the current paths under high strains, the microprism structures enhance sensitivity by concentrating strains in the valley regions. Using one-dimensional self-assembled π-conjugated poly(3-hexylthiophene-2,5-diyl) nanofibrils (P_3_HT-NFs) percolated in a PDMS elastomer matrix (P_3_HT-NF/PDMS) as the rubbery semiconductor nanocomposite in one layer and AgNW/PDMS as the interconnection in another layer, a rubbery strain sensor was constructed, as shown in Figure 10a,b [99]. The AgNW/PDMS composite acts as a stretchable conductor prepared by drop-casting AgNWs on a glass and then spin-coating with PDMS solution. A 3 × 3 strain sensor array, as shown in Figure 10c, was developed to verify its strain-sensing capabilities. There is a change in electrical resistance depending on the strain, as shown in Figure 10d. By incorporating the sensors into a rubber glove, as shown in Figure 10e, various hand gestures can be detected, including finger and wrist bending.

**Figure 11:**
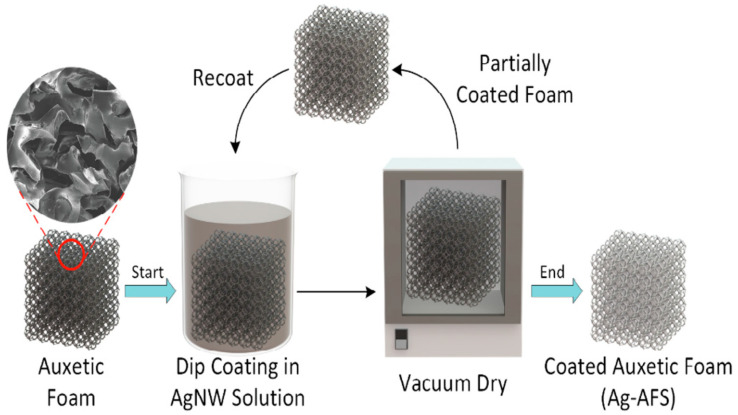
Schematic of the AgNW foam sensor fabrication process. Reprinted with permission from Ref. [101]. Copyright 2019, Elsevier.

Xin Jing et al. [100] formed a hydrogel composite of AgNWs and gelatin, where AgNWs form electrically conductive pathways and reinforce the hydrogel. The thiol groups introduced to the gelatin molecular chain further establish better interactions between the reinforcing AgNWs and the gelatin molecules. By soaking in Na_2_SO_4_ solution, additional physical cross-links are induced by the salting-out effect to produce a stretchable and conductive composite hydrogel. Conventional foam substrates (CFSs) and porous auxetic foams have also been used to prepare stretchable and compressible sensors with AgNWs as conducting elements [101]. The vacuum-dried foams are dip-coated multiple times in the AgNW suspension, as shown in Figure 11. The AgNW/auxetic foam improved by up to 290% and 165% compared to AgNW/CFS in tension and compression modes. Such porous piezoresistive sensors can potentially be used in sportswear, flow detection media, smart healthcare foams, etc. The AgNW concentration impacted the piezoresistive sensitivity, and the sensors were stable for at least 1000 cycles. They could measure strain in all three orthogonal directions, and the sensor could detect pressure as low as 1.5 kPa. Air pressure detection and underwater sensing are the additional features.

**Figure 12:**
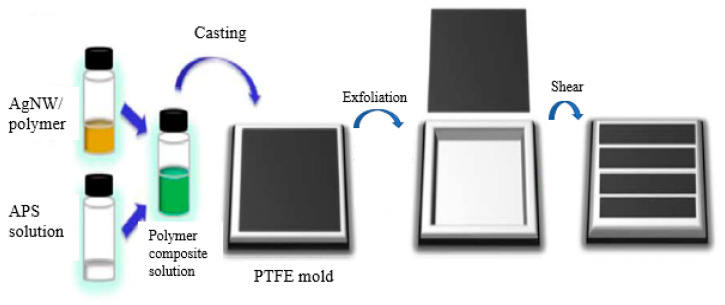
Schematic illustration of the steps in AgNW/polymer composite sensor fabrication. Reprinted with permission from Ref. [103]. Copyright 2020, Springer.

With a high concentration of PVA as a substrate, a conductive hybrid layer of PVA/AgNWs is deposited on it so that the designed bilayer functions as a hydrogel strain sensor [102]. In this new bilayer design, the bottom layer is made of highly concentrated PVA, and the top layer is made of dilute PVA so that AgNWs can be dispersed to form conducting paths. The PVA and AgNW concentrations determine the mechanical properties and the sensing performance. High stability, low hysteresis, and biocompatibility make them suitable for wearable biomedical applications. A mixture of polyacrylic acid, phytic acid solution, aniline, AgNW solution, and ammonium persulfate solution (APS) is molded into a strain sensor using a PTFE mold, as shown in Figure 12. This new variety of polymer increases the stretchability by up to 500% [103].

Among other substrates to pair with AgNWs, cellulose nanofibril (CNF) paper was also tested to prepare stretchable strain sensors [41]. Solution blending and filtration techniques were used to prepare nanofibril solutions and the AgNW/CNF hybrid paper. AgNW homogeneously disperses in the CNF owing to the latter’s amphiphilic property and effectively constructs electrically conductive networks. A TPU-sandwiched AgNW/CNF hybrid paper tensile strain sensor with a microcrack structure displayed a GF of 34.06. A tunable biaxial strain sensor with the ability to respond to structural vibrations and impacts was reported by Robert Herbert et al. using aerosol jet printing of polyimide and silver nanowires [104]. The resistance change is due to the separation and alignment of individual AgNWs, followed by a decrease in the number of junctions along the conductive pathways. The multilayered structures enable better adhesion and lamination on different surfaces. GFs of 1–7.5, stretchability of 4%, and stability of more than 100 cycles of stretching and bending were observed. Aerosol jet printing is a better option for patterning miniaturized stretchable strain sensors. The printing parameters and design variations determine the initial resistance, sensitivity, and strain range.

Facile, low-cost, and scalable fabrication techniques are in great demand in addition to the high stretchability and sensitivity of strain sensors. A biocompatible AgNW/Ecoflex-based composite strain sensor was reported by R. Madhavan [30] using the inkjet printing technique, where functional materials are precisely deposited in a rapid and non-contact approach suitable for high-volume production. Silver nanomaterial deposition with inkjet printing was attempted for the first time. Full contact, a conductive tunneling junction within a cut-off distance, and the complete disconnection of AgNW particles are the three possible situations among AgNW particles. In another instance, extrusion-based 3D printing was used to prepare a biocompatible electronic ink using a copolymer called ω-pentadecalactone-co-ε-decalactone (PDL) and AgNWs [44]. The composite had a low percolation threshold of 1% *w*/*w* AgNWs to PDL and low resistance and anti-microbial properties. With an average gauge factor of 2.78 ± 0.22, the sensor could sense cyclic physiological strains in a customized in vitro setup for more than three weeks.

Kirigami-like structures are applied to various stretchable devices, such as solar panels, implantable and stretchable bioprobes, and tunable optical gratings. Using high-aspect-ratio AgNWs in kirigami-like structures, highly linear strain sensors with reduced hysteresis can be realized [105]. The vacuum-filtered AgNW film on filter paper was patterned using a Silhouette Curio machine. Then, the Ecoflex precursor was spin-coated onto the patterned AgNW film as a stretchable substrate. After curing, the AgNW–Ecoflex composite was detached from the underlying filter paper. Kirigami-structured strain sensors with long AgNWs show high stretchability, excellent linearity (R^2^ ~ 0.99), and up to 70% strain but less sensitivity (GF~1.6). Furthermore, the kirigami-structured strain sensor shows no cracking after strain testing.

In elastomer-based sandwich structures, the resistance increases after repeated stretch/release cycles as the number of detached conductive nanoparticles increases. To overcome this issue, Zhenhua Yang et al. [39] prepared a PDMS/poly(vinylidene fluoride) (PVDF) electrospun membrane pumped with silver nanowire (AgNW) suspensions through a simple filtration process. The PVDF/PDMS electrospun membranes form a mechanically interlocked structure and provide a supporting medium for the isolated AgNWs. Based on a silver nanowire (AgNW) layer and a hydrogel substrate, a highly flexible skin-like strain sensor was presented recently by Krithika Senthilkumar et al. [106]. As a simple production approach, thermal annealing is used to adjust the gauge factor by producing multidimensional wrinkles and a multilayer conductive network. The developed AgNW–hydrogel (AGel) sensors have a stretchability of 200% and a max. GF of 70. Table 5 shows the details of stretchable strain sensors based on AgNW/other substrate composites.

### 7.2. AgNWs as Additional Filler in Other Substrates

**Figure 13:**
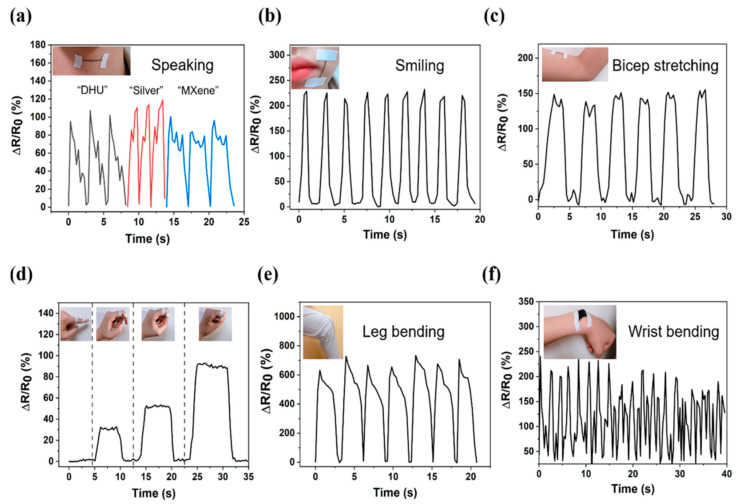
Monitoring of human motion using the nanocomposite MXene Silver (NMS) yarn strain sensor. (**a**) Speaking different words, (**b**) smiling, (**c**) bicep stretching, (**d**) finger bending, (**e**) leg bending, and (**f**) wrist bending. Reprinted with permission from Ref. [109]. Copyright 2019, American Chemical Society.

AgNWs, along with other conducting materials, add to the sensing characteristics. Highly stretchable conductive fibers consisting of AgNWs, AgNPs, and poly(styrene-block-butadiene-block-styrene) (PSBS) polymer were reported by Seulah Lee et al. [31]. The AgNW-mixed SBS fiber was made utilizing an AgNW-dispersed SBS solution dope and a simple wet-spinning process. Wet-spun AgNW-mixed SBS fiber absorbed an AgNP precursor, which was then transformed into AgNPs inside and on the fiber’s outermost surface. The AgNWs aligned with the imposed uniaxial strain and were able to join the unconnected AgNP networks. The implanted AgNWs operate as conducting bridges between AgNPs during stretching, preserving electrical conductivity even under high strain. Silver nanomaterials are mixed with 2D materials such as MXene to improve conductivity [109]. The 0D AgNPs are flexible and act as connections between 1D AgNWs and 2D MXene. With 1D AgNWs significantly improving the conductivity of the strain sensor, 2D MXene (Ti_3_C_2_T_x_), a pliable, flexible graphene-like material, enhances the malleability of the strain sensor. The composite yarn strain sensor with a considerable strain range and sensitivity can effectively monitor various human body movements, as shown in Figure 13.

**Figure 14:**
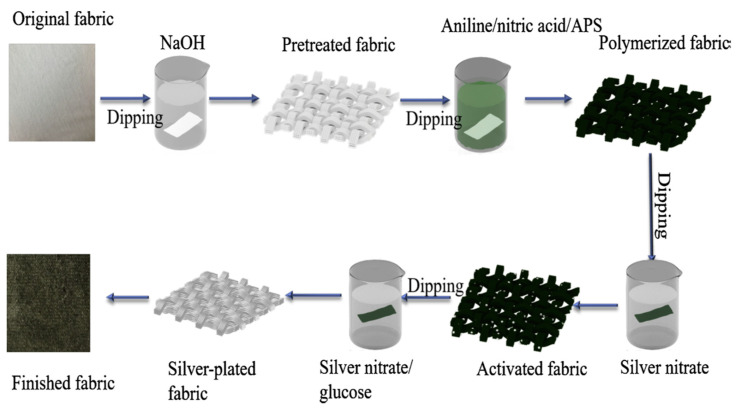
The preparation process of silver-plated fabric. Reprinted with permission from Ref. [33]. Copyright 2019, Elsevier.

As shown in Figure 14, a multifunctional textile-based electronic device with coupled strain-sensing and heating capabilities was produced using silver nanowire/wrap yarn [108]. The wearable electronic device has potential applications in health tracking and thermotherapy. Electroless silver plating is a low-cost and easy-to-process method for metallization. A cotton/spandex blended fabric (95% cotton and 5% spandex) was electroless silver-plated in the work of Zhihua Ma et al. to fabricate a wearable and anti-bacterial strain-sensing fabric that showed a gauge factor of 26.11, a response time of 0.04 s, and a recovery time of about 0.08 s [33].

The fabric is washed with sodium hydroxide solution, in situ polymerized with PANI, and then electroless silver-plated using silver nitrate solution. The breaking strength of the fabric after electroless silver plating is 8.42 MPa, and the elongation at break is 149.89%. PANI has been used to improve the strength between the silver layer and the fabric. By sandwiching a layer of AgNW-decorated self-healing polymers between two layers of PDMS, a flexible 3D architecture was fabricated [43]. Empol 1016 Dimer Acid and diethylenetriamine were used to create the self-healing polymer, and chopped carbon fibers were used to reinforce it. Drop casting was used as the fabrication method to obtain a GF of 1.5 and a stretchability of 60%. The resulting polymer composite was tested for motion monitoring of bending and recovering of various joints. In the work of Yang Liu et al. [110], a self-healing strategy to boost both sensitivity and stretchability was discussed. A resistive-type strain sensor was realized with graphene oxide (GO) nanosheets as the inorganic matrix and AgNWs as the conductive networks on GO nanosheets. Both AgNWs and GO form a conductive brittle nanocomposite with a multi-level nanostructure. Polyvinyl alcohol (PVA)-Cyclo-dextrin (CD) and PVA-adamantane (AD) act as bridging materials for in situ repairs of the cracks and damages induced by structural deformations. The sensor was able to withstand more than a million stretch–release cycles in addition to a stretchability of 58% and a gauge factor of 1591. In another instance, reduced graphene flakes and AgNWs were used as fillers on a spandex fibrous substrate by Tan Thong Vo et al. to realize a stretchable strain sensor [111]. It was identified that the increase in a single filler alone results in reduced stretchability. Hence, an optimal ratio of fillers has to be maintained for robust conductive paths.

Using a self-healing elastomer based on Diels–Alder (DA) bonds, an MXene/AgNW electronic sensor with a multi-scale conductive layer structure was reported by Lun Zhang et al. [112]. The elastomer displayed a self-healing efficiency of more than 88% through the variable density of crosslinkers. Two self-healing elastomer layers sandwiched the conductive MXene and AgNW layers. The AgNWs partially penetrated the elastomer substrate and maintained the conductive paths, while the robust 2D MXene was tightly covered on the AgNW network by the capillary effect. These two nanomaterials enhance the mechanical strength and toughness and hence the stability of the strain sensor. Due to the brittle nature of the layered structures formed by MXene and AgNWs, cracks form and propagate throughout the sensing film, changing resistance to stretching. Further, the dynamic cross-linked network of the elastomer heals the crack cuts upon heating. Nevertheless, the flexible sensors can detect pressure in the range of 183–2260 kPa. The research findings of AgNWs as additional filler in various other substrates are shown in Table 6.

## 8. Conclusions

AgNW composites, with their flexibility, electrical conductivity, transparency, solution processability, and cost-effectiveness, offer equally satisfactory sensing characteristics compared to other fillers. Variations in fabrication steps and novel substrate materials are experimented with to achieve a balanced improvement of sensitivity and stretchability. Hybrid fillers help in achieving an overall improvement in all metrics. Transparency and surface resistance decrease with an increase in AgNW concentration. The intrinsic piezoresistive effects, electron conduction mechanisms, materials selection, and structure design determine the strain-sensing performance.

Specific trends can be noted when looking at recent developments in stretchable resistive strain sensors using AgNW composites. Multifunctional sensors that measure additional parameters such as temperature and pressure are actively explored. Biocompatibility is expected with other features of stretchable strain sensors. High sensitivity, an extensive linearity range, a wide sensing range, high durability, greater tensile strength, and more negligible hysteresis are the main requirements of any flexible and stretchable strain sensor. Other expectations are straightforward preparation methods, cost-effectiveness, large-scale manufacturing, and simpler attached sensor circuitry. Additional features such as self-healing ability, hydrophobicity, transparency, and self-powering can be expected of these sensors in the coming days. Using near-field communication with a mobile phone, the sensor circuit can deliver information such as pulse rate, neck posture, and other human joint motions. Most of the reported strain sensors were tested for their applications in human motion monitoring. Joint motions (wrist, elbow, and finger), respiration and pulse monitoring, eye blinking, sign language through finger bending, and recognition of phonetical expressions are made possible with strain sensors.

## Figures and Tables

**Table 1:** An overview of stretchable strain sensors fabricated using AgNWs as the only filler in the PDMS composite.

Author and Year	Materials (Conducting Elements/Polymer)	Max. Sensitivity	Fabrication Method	Stretchability (%)	Durability (No. of Cycles)	Features
Morteza Amjadi et al., 2014 [63]	AgNW/PDMS	14	Drop casting	70	1000	➢ Bending angle sensitivity: 0.63 rad^−1^ ➢ Linearity: R^2^ = 0.96
Kyun Kyu Kim et al., 2015 [71]	AgNW/PDMS	20	Vacuum filtration and transfer	35	1000	➢ Bi-directional sensing ➢ Simultaneous measurement of spatial strain distribution
Xinqin Liao et al., 2017 [72]	AgNW/PDMS	150,000	Coating Pre-stretching Drying	60	30,000	➢ Microcrack-assisted resistive strain sensor ➢ Low creep ➢ Useful in smart wearable systems
Chan-Jae Lee et al., 2017 [67]	AgNW/PDMS	30	Spin coating	100	2500	➢ Transparency: >90% at 550 nm wavelength
Yi Du et al., 2018 [73]	AgNW/PDMS	536.98	Drop casting	9	-	➢ Stable over a wide temperature range ➢ Useful in pulse sensing
Ji Hwan Cho et al., 2018 [36]	AgNW microwire grid/PDMS	41.1	Dip coating Spin coating Mesh-template-assisted contact transfer printing	35	1000	➢ Useful in human motion monitoring ➢ Optical transparency: 77.1 ± 1.5% ➢ Negligible hysteresis
Wei Li et al., 2019 [58]	AgNW/PDMS	4.11	Semi-dry method	-	200	➢ Useful in elderly care applications ➢ Low hysteresis ➢ Optimized parameters for rapid polyol method
Zhihui Wang et al., 2019 [48]	AgNW/PDMS	846	Laser cutting Drop coating	150	1000	➢ Transparency: 88.3% ➢ Tested for monitoring subtle and large motions
Fanqi Yin et al., 2019 [69]	AgNW/PDMS	84.6	Water-bath pulling method	40	10,000	➢ Transparency: 86.3%
Pegah Hashemi et al., 2020 [56]	AgNW/PDMS	8.32	Casting	54	-	➢ Medical applications ➢ Sandwich-like structure
Jae Hyuk Choi et al., 2020 [70]	AgNW/PDMS	89.99	Dip coating	60	1000	➢ Sandwich structure ➢ Negligible hysteresis
Wei-Wei Kong et al., 2021 [27]	AgNW/PDMS	3	Spray coating plus rolling	100	6000	➢ Negligible hysteresis ➢ Linearity: 0.99

**Table 2:** An overview of recent research works relating to AgNWs as an additional filler in the strain-sensing PDMS composite.

Author and Year	Materials (Conducting Elements/ Polymer)	Max. Sensitivity	Fabrication Method	Stretchability (%)	Durability (No. of Cycles)	Features
Wei Hu et al., 2016 [82]	AgNW and PEDOT:PSS/PDMS	15	Spin coating	20	1000	➢ Transparency: 80% ➢ Useful in real-time monitoring of neck-posture ➢ Thickness: 20 μm ➢ Response time: 20 ms ➢ Recovery time: 40 ms
Lihua Liu et al., 2017 [74]	AgNW and AgNP/PDMS	547.8	Heat polyols thermal method	7.26	-	➢ Useful in flexible electronics ➢ Low production cost ➢ Sandwich structure
My Duyen Ho et al., 2017 [75]	AgNW and AuNW/PDMS	236	Drop casting Langmuir–Blodgett transfer technique	70	1000	➢ Transparency: 58.7 to 66.7% ➢ Low detection limit: 0.05% ➢ Operating voltage: 0.1 V
Shengbo Sang et al., 2018 [53]	AgNP and AgNW/PDMS	3766	Drop casting in template method	28.1	-	➢ Useful in body movement testing ➢ Linear region: 0–28.1%
Shasha Duan et al. [76] 2018	AuNW and AgNW/PDMS	2370	Electrospinning Spin coating	90	1000	➢ Useful in transparent and stretchable electrodes ➢ Transmittance: 86%
Xi Fan et al., 2018 [83]	AgNW and PEDOT:PSS/PDMS	8	Spin coating	50	300	➢ Electrical conductivity: 3100 S/cm ➢ Tested for monitoring finger motions
Jinjin Luan et al., 2019 [77]	Ag film and AgNW/PDMS	21.1	Dip coating, Vacuum thermal evaporation	30	1000	➢ Tested for monitoring joint movements and subtle motions of the mouth
Gui-Shi Liu et al., 2020 [79]	AgNW-acrylate/PDMS	10,486	Spin coating, UV exposure, ultra-sonication	20	10,000	➢ Transparency: 90.3% ➢ Accurate monitoring of pulses and motions
Guishan Wang et al., 2020 [80]	AgNW and graphene/PDMS	1403.7	Vacuum filtration	35	500	➢ Response time: <10 ms ➢ Potential applications in wearable devices and soft robotics
Gengzhe Shen et al., 2020 [84]	AgNW and PEDOT:PSS/ PDMS	10.2	Near-field electrospinning	100	2000	➢ Useful in sensing temperature, HMI, e-skin, and wearable devices ➢ Transparency: 68.3–61.4%
Shicong Niu et al., 2021 [81]	AgNW and graphene/PDMS	9156	Drop coating	60	500	➢ Low hysteresis and resilience above 94%
Meng Yang Liu et al., 2022 [85]	AgNW and CNT/PDMS	6.7	Spray coating Spin coating	50	1000	➢ Response time: 420 ms ➢ Recovery time: 600 ms

**Table 3:** An overview of recent stretchable strain sensors made of AgNW/TPU composite.

Author and Year	Materials (Conducting Elements/ Polymer)	Max. Sensitivity	Fabrication Method	Stretchability (%)	Durability (No. of Cycles)	Features
Lijun Lu et al., 2017 [89]	AgNW/TPU and PDMS	12.9	Electrospinning Vacuum filtration Spin coating	50	1600	➢ Tested for human motion detection ➢ Electrical conductivity: 50 S cm^−1^
Runfei Wang et al., 2019 [88]	AgNW/TPU	337	Rod coating Dip coating	85	2000	➢ Response time: 10 ms ➢ Transparency: 91%
Wei Pan et al., 2020 [29]	AgNW/TPU	44.43	Electrospinning Dip Coating	900	20,000	➢ A conductivity of 3990 S/cm ➢ Spring-like configuration ➢ Useful in finger motion and knee motion sensing, etc.
Pegah Hashemi et al., 2020 [56]	AgNW/TPU	6.78	Casting	372	-	➢ It can be used in medical applications ➢ Sandwich-like structure
Yan Li et al., 2020 [90]	AgNW/TPU	4.4 × 10^7^	Spray coating	100	1000	➢ Tunable sensitivity and stretchability ➢ Tested for human motion monitoring

**Table 4:** Overview of a few research works on AgNW/PU-based stretchable strain sensors.

Author and Year	Materials (Conducting Elements/ Polymer)	Max. Sensitivity	Fabrication Method	Stretchability (%)	Durability (No. of Cycles)	Features
Byeong-Ung Hwang et al., 2015 [96]	AgNW and PEDOT:PSS/PU	12.4	Blending Spin coating	100	1000	➢ A self-powered patchable strain-sensing platform ➢ Transmittance: 75.3%
Conor S. Boland et al., 2017 [97]	AgNW/PU	70	Layer-by-layer vacuum filtration	250	500	➢ Thickness: 50 μm ➢ Electrical conductivity: 10^4^ Sm^−1^
Guan-Jun Zhu et al., 2019 [28]	AgNW/PU	87.6	Capillary tube method	43	2500	➢ Response time: 49 ms ➢ Conductivity: 3.1 S/cm ➢ Fiber strain sensor with a millimeter diameter ➢ High elongation at break: 265%
Yi Xi Song et al., 2019 [59]	AgNW/PU	11.2	Spray coating Plasma treatment	500	5000	➢ Response time: 200 ms ➢ Self-healability and transparency
Jun Hong Pu et al., 2019 [94]	AgNW/WPU	1.6 × 10^7^	Layer-by-layer dip coating	100	1000	➢ Response time: 344 ms ➢ Relaxation time: 344 ms ➢ Negligible hysteresis ➢ Tensile strength: 15 MPa ➢ Elongation at break: 800%
Fengchao Li et al., 2020 [95]	MXene, AgNW and PEDOT:PSS/PU	1933.33	Screen printing	60	1000	➢ Temperature-sensing ability with a resolution of 0.2 °C ➢ Four linear regions with linearities above 0.972
Yu Jiang et al., 2021 [98]	AgNW and PANI/PU	59	Electrospinning Vacuum filtration	35	300	➢ Electrical conductivity: 32.09 S/m ➢ Tested for human motion monitoring

**Table 5:** Overview of stretchable strain sensors using AgNWs as a single filler in other substrates.

Author and Year	Materials (Conducting Elements/ Polymer)	Max. Sensitivity	Fabrication Method	Stretchability (%)	Durability (No. of Cycles)	Features
Seongcheol Mun et al., 2016 [107]	AgNW/cellulose film	4.3	Layer-by-layer spray coating	2	-	➢ Transmittance: 70% ➢ Tested in stretching and bending modes
K H Kim et al., 2018 [32]	AgNW/Dragon Skin	81	Soft lithography Drop casting	150	10,000	➢ Useful in human motion monitoring ➢ Pressure sensitivity: 3 Pa^−1^ ➢ Linearity: R^2^ = 0.993
Hae Jin Kim et al., 2018 [99]	AgNW/PDMS/P_3_HT-NF/PDMS	32	Drop casting Spin coating	100	-	➢ Hysteresis: 12% ➢ Linearity: R^2^ > 0.996
Min Zhao et al., 2019 [108]	AgNW/cotton yarn and PU monofilament	4.2	Dip coating	200	1000	➢ Ability to serve as a heater ➢ Resistance: 36 Ωcm^−1^
Shohreh Azadi et al., 2019 [102]	AgNW/PVA	0.58	Freezing thawing	500	2000	➢ Hysteresis: 7% ➢ Response time: 0.32 s ➢ Mechanical strength: 900 kPa ➢ Electrical conductivity: 1.85 Sm^−1^
Xin Jing et al., 2019 [100]	AgNW/gelatin	2.4	Dispersion and solution casting	200	-	➢ Conductivity: 0.1 S/cm ➢ Can be applied in biosensors, e-skin, and health monitoring devices
Rui Yin et al., 2020 [41]	AgNW/CNF	34.06	Solution blendingVacuum filtration	2	500	➢ Low detection limit: 0.2% ➢ Temperature sensing ability ➢ Tension mode GF: 10.2 ➢ Compression mode GF: 1.2
Yanjing Zhang et al., 2020 [103]	AgNW, aniline, phytic acid, polyacrylic acid and APS composite	2.2	Solution casting	500	1000	➢ Low detection limit: 1% ➢ Useful in human motion and healthcare monitoring ➢ Conductivity: 1.3 S/m
R. Madhavan 2021 [30]	AgNW/Ecoflex	13.7	Inkjet printing	30	1000	➢ Low detection limit: <5% ➢ High linearity (R^2^ > 0.98)

**Table 6:** Overview of stretchable sensors using AgNWs as additional filler in other substrates.

Author and Year	Materials (Conducting Elements/ Polymer)	Max. Sensitivity	Fabrication Method	Stretchability (%)	Durability (No. of Cycles)	Features
Seulah Lee et al., 2015 [31]	AgNW and AgNP/PSBS	15	Wet spinning	220	1000	➢ Max. elongation at break: 900% ➢ Electrical conductivity: 2450 S/cm
Songjia Han et al., 2018 [113]	AgNW and PEDOT:PSS/Ecoflex	2000	Deposition, Spin coating, Injection	420	3000	➢ Tested for human–machine interactive systems ➢ Potential bionic ligaments in soft robotics
Zhihua Ma et al., 2019 [33]	AgNW and PANI/cotton/spandex	26.11	Electroless silver plating	4	>160	➢ Electrical conductivity: 15.7 S/m ➢ Low detection limit: 0.2%
Han Li and Zhaoqun Du 2019 [109]	AgNP, AgNW, and MXene/Dacron fibers	872.79	Mixing Dipping Drying	350	1500	➢ Useful in intelligent textiles
Tan Thong Vo et al., 2020 [111]	AgNW and rGO/spandex	150.3	Dropcasting	120	1000	➢ Electrical conductivity: 8.06 S/m ➢ Low hysteresis
Kittiphong Thana et al., 2021 [114]	AgNW and PEDOT:PSS/natural rubber	418	Spin coating	50	750	➢ High stability for rapid bending ➢ Low limit of detection: 3.5% ➢ Electrical resistance: 74.72 ± 14.65 Ω
Yanqiang Cao et al., 2021 [115]	AgNW and MWCNT/hair band	416	Dipping	70	7500	➢ Facile, low-cost, and scalable production
Liangjun Chen et al., 2021 [116]	AgNP and AgNW/latex balloon	2.8 × 10^5^	Spray coating	80	1000	➢ Tested for both microstrains and large strains
Lun Zhang et al., 2022 [112]	AgNW and MXene/a self-healing elastomer	>29.4	Spraying	96	1200	➢ Self-healing efficiency: 88% ➢ Response time: ~71 ms ➢ Relaxation time: ~138 ms ➢ Pressure sensing ability

## Data Availability

Not applicable.
